# Development of a Machine Learning Model for the Classification of *Enterobius vermicularis* Egg

**DOI:** 10.3390/jimaging10090212

**Published:** 2024-08-28

**Authors:** Natthanai Chaibutr, Pongphan Pongpanitanont, Sakhone Laymanivong, Tongjit Thanchomnang, Penchom Janwan

**Affiliations:** 1 Medical Innovation and Technology Program, School of Allied Health Sciences, Walailak University, Nakhon Si Thammarat 80160, Thailand; natthanai.c@phuket.psu.ac.th; 2 Hematology and Transfusion Science Research Center, Walailak University, Nakhon Si Thammarat 80160, Thailand; 3 Medical Technology Service Center, Prince of Songkla University, Phuket Campus, Phuket 83120, Thailand; 4 Health Sciences (International Program), College of Graduate Studies, Walailak University, Nakhon Si Thammarat 80160, Thailand; pongphan.po@wu.ac.th; 5 Centre of Malariology, Parasitology and Entomology, Ministry of Health, Vientiane Capital P.O. Box 0100, Laos; sakhone07@gmail.com; 6 Faculty of Medicine, Mahasarakham University, Maha Sarakham 44000, Thailand; tongjit.t@msu.ac.th; 7 Department of Medical Technology, School of Allied Health Sciences, Walailak University, Nakhon Si Thammarat 80160, Thailand

**Keywords:** *Enterobius vermicularis*, deep learning, machine learning, computer vision, object detection

## Abstract

*Enterobius vermicularis* (pinworm) infections are a significant global health issue, affecting children predominantly in environments like schools and daycares. Traditional diagnosis using the scotch tape technique involves examining *E. vermicularis* eggs under a microscope. This method is time-consuming and depends heavily on the examiner’s expertise. To improve this, convolutional neural networks (CNNs) have been used to automate the detection of pinworm eggs from microscopic images. In our study, we enhanced *E. vermicularis* egg detection using a CNN benchmarked against leading models. We digitized and augmented 40,000 images of *E. vermicularis* eggs (class 1) and artifacts (class 0) for comprehensive training, using an 80:20 training–validation and a five-fold cross-validation. The proposed CNN model showed limited initial performance but achieved 90.0% accuracy, precision, recall, and F1-score after data augmentation. It also demonstrated improved stability with an ROC-AUC metric increase from 0.77 to 0.97. Despite its smaller file size, our CNN model performed comparably to larger models. Notably, the Xception model achieved 99.0% accuracy, precision, recall, and F1-score. These findings highlight the effectiveness of data augmentation and advanced CNN architectures in improving diagnostic accuracy and efficiency for *E. vermicularis* infections.

## 1. Introduction

*Enterobius vermicularis*, commonly known as pinworm, affects approximately 200 million people worldwide, significantly impacting children [1]. This intestinal infection, known as enterobiasis, poses a major public health issue globally and in Thailand [1,2]. Infection typically occurs through ingestion of embryonated eggs from contaminated surfaces, food, or fingers. The unique epidemiological and socio-economic conditions in Thailand exacerbate the problem, with communal living environments in schools and rural areas facilitating the rapid spread of *E. vermicularis*. Limited access to proper sanitation and hygiene facilities further intensifies the transmission, creating substantial public health challenges. Children are particularly vulnerable due to their close contact and play behaviors, often suffering from symptoms such as perianal itching, sleep disturbances, and nutritional deficiencies, which can impair growth and cognitive development, affecting their education and future opportunities. Additionally, the economic impact of reduced productivity and school attendance due to illness, along with healthcare expenses for diagnosis and treatment, strains the healthcare system and the national economy [3]. The definitive diagnosis of *E. vermicularis* relies on conventional methods such as the adhesive tape test (also known as the scotch tape test), which is considered the most effective tool due to the female worm’s nocturnal egg-laying behavior on the perianal skin [4]. In the adhesive tape test, a piece of clear adhesive tape is pressed against the perianal region early in the morning before bathing or using the toilet, then placed on a microscope slide for examination to identify the characteristic eggs of *E. vermicularis*. This method, while simple and cost-effective, is labor-intensive and requires significant expertise. In busy clinical microscopy services, relying on qualified examiners for adhesive tape tests is time-consuming and prone to human error from exhaustion and cognitive stress. As a result, infected individuals may develop ongoing symptoms that can significantly affect the patient’s quality of life, including sleep disturbances and social discomfort. Continued symptoms without a proper diagnosis may lead to further medical consultations and investigations, increasing healthcare costs and burden on the system, and an increase in the number of community members who can be carriers, resulting in high-risk regions.

With advancements in computer vision technologies, the parasitic detection process has been significantly enhanced [5,6]. Modern systems employ deep learning (DL) algorithms, particularly convolutional neural networks (CNNs), which are highly effective in image analysis tasks. These artificial intelligence (AI)-driven models are trained on extensive datasets of labeled images, including various stages of parasitic eggs and larvae, captured through high-resolution digital microscopy. Recent applications in parasitic infections have emerged as crucial areas for the application of AI and DL, significantly enhancing diagnostic accuracy and efficiency. Traditional methods of diagnosing parasitic infections, such as manual microscopy, are labor-intensive and require highly skilled personnel, which poses challenges, especially in resource-limited settings [7,8]. Advancements in AI have led to the development of various object detection models tailored for identifying parasitic pathogens in microscopic images. For instance, the You Only Look Once version 3 (YOLOv3) model was utilized to detect three different types of helminth eggs. The model demonstrated excellent performance, accurately identifying eggs of *Schistosoma* spp., *Ascaris* spp., and *Trichuris* spp. The sensitivities achieved for detecting these eggs were 95.31%, 86.36%, and 80.00%, respectively, displaying the model’s capability to handle the complexities of egg morphology and variations within the dataset [9]. The tiny version of YOLO version 4 (YOLOv4-Tiny) model has shown remarkable performance in detecting protozoan cysts and helminthic eggs in human feces, achieving a precision of 96.25% and a sensitivity of 95.08% [7]. Similarly, an ensemble approach combining FasterRCNN, Task-aligned One-stage Object Detection (TOOD), YOLOX, and Cascade with Swin Transformers has demonstrated superior performance with an intersection over union (IoU) of 0.915, outperforming individual models [10]. In the context of malaria diagnosis, YOLOv5 has been identified as the most effective model among several evaluated, demonstrating high precision and sensitivity in detecting *Plasmodium* spp. in blood smear images [11]. To the best of our knowledge, our study is the first to demonstrate *E. vermicularis* egg detection and classification by using machine learning (ML). For the diagnosis of enterobiasis, the process begins with the collection of scotch tape tests or stool samples, which are then digitized using advanced imaging techniques. These images are fed into the computer vision system, where preprocessing steps such as image normalization, noise reduction, and contrast enhancement are applied to improve the clarity and quality of the images. The CNNs then analyze these preprocessed images, identifying and classifying the morphological features of the *E. vermicularis* egg with remarkable accuracy. Key features such as the oval shape of the eggs, the presence of a clear, colorless shell, and the characteristic larvae within the eggs are detected and highlighted by the AI. Additionally, the models are designed to differentiate between *E. vermicularis* and other similar-looking parasites or artifacts, reducing the likelihood of false positives. One of the significant advantages of this transition to computer vision is the scalability and efficiency it offers. Automated systems can process large volumes of samples in a fraction of the time required for manual examination, making them ideal for large-scale screening programs. Furthermore, the consistent performance of AI models minimizes the variability and subjectivity inherent in human analysis, leading to more reliable and reproducible results. This approach has the potential for a worldwide impact, significantly contributing to the global fight against enterobiasis.

## 2. Materials and Methods

### 2.1. Sample Preparation and Image Capturing

Sample collection using scotch tape techniques: The scotch tape technique was instrumental in gathering samples, especially for *E. vermicularis*. This method is well known for its effectiveness in collecting eggs from the perianal area, offering a clear view under the microscope. This approach enabled robust sample collection for our study. To collect samples using the scotch tape method, we focused on children aged 3 to 10 years. Before starting, we prepared and quantified all the necessary materials, such as glass slides and transparent adhesive tape, based on the number of volunteers. At the designated community site, we established a private and controlled setting. Researchers donned protective gear, including hairnets, masks, and gloves, to prevent contamination. The collection process involved pressing a piece of clear tape, roughly 2 cm wide and 6 cm long, against the child’s anal area for a few seconds. This tape, potentially containing samples, was then adhered to a glass slide. After collection, the slides were analyzed under a light compound microscope in a laboratory and could be stored at room temperature for up to three weeks.

Concurrently, we developed an ML model to classify helminth eggs. Data for this model were primarily gathered using the scotch tape method, focusing on parasites such as *E. vermicularis*. High-resolution images of the helminth eggs were taken with an advanced Olympus BX43 + DP27 microscope (Olympus, Tokyo, Japan) at 400× at the Parasitology Laboratory, Walailak University. After capturing the images, each was systematically cataloged and organized into specific categories. The medical technologists from our research team supervised and confirmed the identification of *E. vermicularis* egg type to ensure accuracy. The images, with a resolution of 2448 × 1920 pixels, were stored in Tagged Image File Format. A total of 2000 images were collected.

This study’s protocol was approved by the Ethics Committee in Human Research at Walailak University, based on the Declaration of Helsinki (WUEC-23-051-02). Our study’s purpose and procedures were explained to the participants prior to enrollment. The aim and all processes of this study were fully described to the children and parents or legal guardians, and participation was voluntary. Written informed consent was obtained from a parent or guardian on behalf of any participants before data and sample collection. All study participants infected with *E. vermicularis* were treated with mebendazole.

### 2.2. Model Training and Image Recognition

#### 2.2.1. Data Preparation

**Figure 1:**
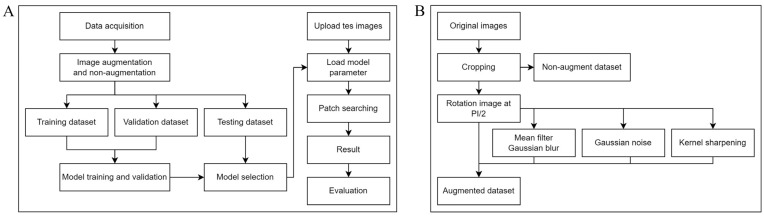
The workflow of an object detection system incorporating data augmentation techniques is illustrated. (**A**) This comprehensive process begins with data acquisition, followed by preprocessing and image augmentation. The augmented images are used for model training, after which the model undergoes validation and testing. Once trained, the model is applied to new test images to detect objects, with performance evaluated using the Intersection-over-Union (IoU) metric. (**B**) Various augmentation techniques are applied to the original dataset, creating an enhanced and diversified dataset.

Dataset preprocessing: auto-cropping: The objective was to standardize the dimensions of all images and focus on *E. vermicularis* eggs. Images were carefully cropped to a size of 370 × 370 pixels, ensuring the essential features of the parasitic eggs were preserved. A total of 1000 images per class, amounting to 2000 images, were prepared. Class 0 refers to artifacts, which are objects or items that might be mistaken for eggs but are irrelevant elements found in the images. Artifacts can include any non-egg particles or debris collected during the scotch tape technique used to prepare the slides. Class 1 refers to *E. vermicularis* eggs, identifying the actual parasitic eggs needed for accurate diagnosis of enterobiasis. Our training dataset, sourced from the scotch tape technique, included 2000 images with an equal 1:1 ratio between *E. vermicularis* eggs (class 1) and artifacts (class 0). Artifacts such as air bubbles, plant cells, clothing fibers, and other non-parasitic entities were included to train the model in distinguishing actual parasitic eggs from common confounders in scotch tape test examinations. For consistency, all images were resized to 370 × 370 pixels. Bicubic interpolation was used to ensure quality and feature preservation during resizing. Special care was taken to maintain the integrity of visual information, which is crucial for the successful training and testing of the CNN model. By carefully preserving essential features in the images and curating a balanced dataset with various *E. vermicularis* egg and artifact images, we established a solid foundation for the model’s learning and detection processes. With the data acquisition phase thoroughly conducted (Figure 1A), we are now ready to proceed to the next step in our research methodology: data augmentation (Figure 1B). This phase will involve using various techniques to further enrich our dataset, enhancing the model’s ability to generalize and accurately identify *E. vermicularis* eggs under different conditions.

#### 2.2.2. Data Augmentation

Enhancing the diversity and volume of training datasets through image data augmentation is a crucial technique widely used in ML and computer vision, particularly when original data are limited [12,13,14,15]. By artificially expanding the dataset with modified versions of existing images, data augmentation helps prevent overfitting and enhances the generalization capabilities of models. Techniques for image data augmentation range from basic geometric transformations, such as rotations, scaling, and flipping, to more advanced color adjustments, including variations in brightness, contrast modulation, and noise injection. These basic transformations are popular due to their simplicity and effectiveness in introducing variability into the data without requiring extensive computational resources [16]. Image data augmentation is a cornerstone technique in AI, crucial for enhancing model training processes. It enables effective scaling of limited datasets and improves data diversity and quality, leading to better model performance and generalization across various tasks.

The data augmentation process (Figure 1B) begins immediately after the image cropping stage. This strategic approach enriches the dataset by introducing various transformations, thereby enhancing its diversity. The primary objective of implementing data augmentation at this stage is to strengthen the ML model’s ability to robustly handle and accurately interpret images with varied orientations. This augmentation is crucial for ensuring consistent model performance across different visual scenarios.

Rotation of images: To further augment the dataset, cropped images across both classes were rotated at 90-degree intervals, creating four distinct versions of each original image. This methodological approach significantly expanded the dataset, resulting in 4000 rotated images per class, totaling 8000 augmented images.

Mean Filtering: This technique was applied to the 8000 rotated images to smoothen the image by reducing random noise and creating a uniform background conducive to accurate analysis. The pixel values within a kernel size of 5 were averaged, resulting in 8000 clearer images.

Gaussian blur: This technique was introduced to the same 8000 rotated images, creating a subtle blurring effect that highlighted the primary features of the *E. vermicularis* eggs while reducing high-frequency noise and correcting uneven illumination. The images were convolved with a Gaussian function with a kernel size of 5, producing another set of 8000 augmented images.

Gaussian noise: Random pixel values based on a Gaussian distribution were introduced to the 8,000 rotated images. This step aimed to test the model’s adaptability to noise and simulate real-world challenges, resulting in 8000 images that tested the model’s robustness against noise.

Sharpening using Laplacian: The Laplacian sharpening technique was applied to the 8000 rotated images to enhance the edges within the microscopic images, ensuring better clarity and distinction of *E. vermicularis* eggs. This technique leveraged the Laplacian filter, producing 8000 images with enhanced intensity changes and emphasized edges.

**Figure 2:**
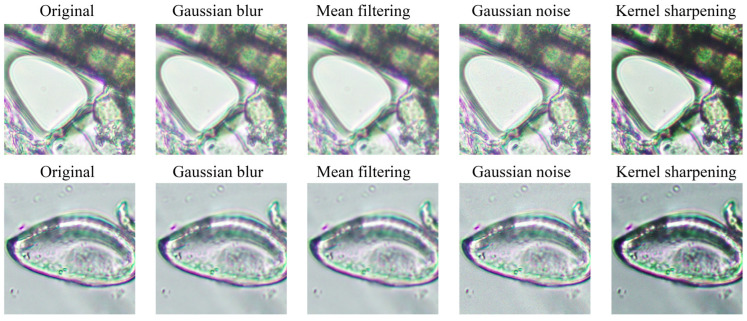
A series of microscopic images illustrating the impact of various image augmentation techniques on two distinct classes (class 0 and class 1). The first column features the original, unaltered images. Moving to the right, each subsequent column reveals the transformed images after applying different augmentation methods: Gaussian blur, mean filtering, Gaussian noise, and kernel sharpening. These techniques introduce a range of visual variations and distortions, enriching the dataset. By incorporating these augmentations, the goal is to bolster the robustness and enhance the generalization capabilities of the machine learning model trained on this enriched dataset. The upper row of images represents class 0, and the lower row represents class 1.

As shown in Figure 2, this multifaceted approach to data augmentation and image processing significantly fortified the dataset’s volume and quality. By applying five distinct image processing features to the initially rotated images, the dataset expanded immensely, resulting in a total of 40,000 images. This comprehensive dataset forms the backbone for training and testing the ML model, enhancing its potential for accuracy and robustness in identifying helminth eggs from microscopic images.

#### 2.2.3. Our Publicly Accessible Dataset of *E. vermicularis* Egg Images

A comprehensive training dataset is provided on Figshare (https://doi.org/10.6084/m9.figshare.26266028.v2, accessed on 22 August 2024) in the folder [01_training_dataset]. This folder contains a variety of images curated for training machine learning models. For model evaluation, images are available in the folder [02_testing_dataset], which includes a diverse set of test images to ensure robust model validation.

For object detection tasks, a collection of original images is located in the folder [03_obj_det_original]. These images are specifically selected to cover a wide range of object types and scenarios. Additionally, expert annotations for the images in [03_obj_det_original] are meticulously compiled in the folder [04_obj_det_expert]. These annotations provide precise labeling to facilitate advanced object detection model training and evaluation.

### 2.3. Model Selection and Comparative Analysis

Besides the primary CNN, various other models were chosen to conduct a comparative performance analysis to ensure the reliability and generalizability of the results. The selected models encompass a wide range known for their effectiveness in image recognition tasks, particularly in medical imaging. The models chosen for the comparison include the following:

Xception: Recognized for its depthwise separable convolutions, which enhance its efficiency and capability in processing complex image data.

MobileNet: Developed for its lightweight structure, making it ideal for scenarios with limited computational resources.

EfficientNetB1: Selected for its scalable design that balances complexity and accuracy.

DenseNet121: Included due to its unique connectivity pattern that improves the information and gradient flow throughout the network, enhancing performance.

InceptionV3: Chosen for its hybrid convolutional architecture, which employs multiple kernel sizes to capture information at various scales.

ResNet50: Incorporated because of its deep residual learning framework, facilitating the training of deeper networks effectively.

These models were selected to provide a comprehensive overview of how various architectures and their distinct features perform in the task of detecting *E. vermicularis* eggs.

#### 2.3.1. Criteria for Comparative Analysis

Evaluating ML models requires a multifaceted approach that considers various criteria, reflecting the diverse capabilities and applications of these technologies. Key metrics and methods for comparing ML performance are derived from extensive research and practical applications, forming a solid foundation for assessing different models across multiple scenarios. A confusion matrix visualizes an ML model’s performance beyond mere accuracy by offering a detailed breakdown of correct and incorrect predictions, categorized into true positives, true negatives, false positives, and false negatives. This helps understand the model’s sensitivity (recall) and specificity, vital for applications where the cost of different errors varies significantly, such as in medical diagnostics or spam detection. The receiver operating characteristic (ROC) curve depicts the diagnostic ability of a binary classifier as its discrimination threshold changes, with the area under the ROC curve (ROC-AUC) providing a single scalar value to measure overall performance across all classification thresholds. This metric is particularly useful for evaluating trade-offs between the true positive rate (sensitivity) and the false positive rate (1-specificity), essential in medical testing where misdiagnosis can have severe consequences. Comparative studies of ML algorithms emphasize the importance of training time, prediction time, and prediction accuracy. These factors are vital in selecting suitable algorithms for specific applications, where the model’s efficiency can significantly impact practical deployment [17]. Metrics such as accuracy and the AUC are frequently used to compare ML algorithms, providing a quantitative basis for assessing and selecting the most effective models. Rigorous comparisons between human capabilities and ML algorithms focus on understanding the differences in cognition and problem-solving approaches, aiding in designing ML systems that complement or enhance human efforts [18]. Additionally, ML models used to predict the execution time of computer programs have shown the influence of static code features, HTTP calls, and hardware performance. Random forest has achieved high accuracy in these predictions, optimizing computational resources [19].

For tasks like medical imaging or object detection, where precise bounding boxes are critical, IoU measures the overlap between predicted and actual bounding boxes, providing a percentage that indicates localization precision. Comparing IoU scores from ML models with those annotated by medical professionals reveals discrepancies in spatial understanding and practical usability in clinical settings. This comparative analysis not only benchmarks the model’s performance against expert annotations but also identifies areas for specific improvements in model training to better align with expert judgment. Incorporating these advanced metrics into performance evaluation frameworks enhances the depth and breadth of comparative studies. By using the confusion matrix, ROC, and IoU, researchers and practitioners gain a detailed understanding of where ML models excel and where they fall short compared to human experts. This aids in fine-tuning the models and provides crucial insights into their operational deployment in real-world scenarios. Adding these criteria makes the framework for evaluating and comparing ML models more robust, addressing both quantitative and qualitative aspects of model performance and ensuring a well-rounded assessment, particularly in applications where precision and reliability are paramount.

#### 2.3.2. A Proposed Convolutional Neural Network Model for *E. vermicularis*

**Figure 3:**
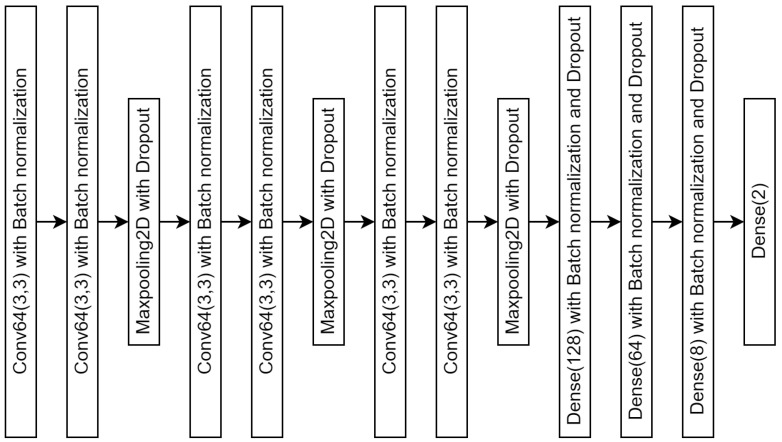
Architectural design of proposed convolutional neural network (CNN).

Our CNN model presented in Figure 3 is structured to perform a binary classification task, as indicated by its final output layer. The architecture begins with a sequence of convolutional layers, each utilizing 64 filters with a 3 × 3 kernel size. These layers are followed by batch normalization, which is implemented to enhance training stability and efficiency by normalizing the activations across mini-batches. After every two convolutional layers, max pooling is applied to downsample the spatial dimensions of the feature maps, thereby reducing computational complexity and emphasizing the most critical features. To further prevent overfitting, dropout is introduced following the max-pooling layers, randomly omitting units during training. The model then progresses from convolutional operations to fully connected layers, comprising three dense layers with 128, 64, and 8 units, respectively. Each dense layer is also followed by batch normalization and dropout to maintain regularization. The final dense layer contains two units, making it appropriate for binary classification tasks. This architecture, characterized by its depth and the integration of multiple regularization techniques, is designed to effectively manage complex data while minimizing the risk of overfitting.

Input: The initial input fed into our model consists of color images with dimensions of 370 × 370 pixels, potentially containing *E. vermicularis* eggs or artifacts. These images are resized to 128 × 128 pixels using bicubic interpolation to meet the network’s requirements, ensuring crucial features for accurate detection are retained.

Convolution layer: The convolution layer applies an array of filters to the input images, generating a feature map that captures essential attributes like edges and textures. This feature map undergoes a non-linear transformation via a Rectified Linear Unit (ReLU) activation function. A pooling operation is used to reduce the spatial dimensions of the feature map, streamlining computational demands. In Figure 3, the model incorporates three successive sets of convolutional and max-pooling layers, with a flattened layer acting as a bridge between the convolutional layers and the fully connected neural networks.

Neural networks: Following the convolutional processes, the feature maps are flattened and fed into a series of fully connected layers. The neural network includes dense layers with 256, 16, and 2 nodes in the output layer, classifying images into categories: *E. vermicularis* egg (class 1) or artifact (class 0), as shown in Figure 3.

Output: The output layer uses a sigmoid activation function to calculate the probability distribution over the two classes, assigning the class with the higher probability to the input image.

Comparative analysis: After establishing the baseline performance of the CNN with both non-augmented and augmented datasets, we conduct a comparative analysis with other prominent DL models: ResNet50, DenseNet121, Xception, MobileNet, InceptionV3, VGG16, and EfficientB1. Each model is evaluated using the augmented dataset to determine its effectiveness in detecting *E. vermicularis* eggs. This approach helps us ascertain whether data augmentation improves model performance and identify which architecture best recognizes and classifies the target objects under varied conditions.

### 2.4. Model Training and Validation

For our model’s training and validation, we leveraged Google Colab Pro^®^ (Google Inc., Mountain View, CA, USA), taking advantage of high-performance hardware typically unavailable in standard setups. Our environment was outfitted with an NVIDIA A100 GPU, an Intel Xeon CPU running at 2.3 GHz, 51 GB of RAM, and 320 GB of storage. This sophisticated configuration provided the necessary computational resources for our rigorous tasks. The experiments utilized the previously described CNN model, with training set to a maximum of 200 epochs. To enhance efficiency and prevent overfitting, an early stopping mechanism terminated the training if the loss fell below 0.001. The optimization process employed the Adam optimizer, with mean squared error as the loss function and a learning rate of 0.001. During training, the model learned to categorize images as either *E. vermicularis* eggs (class 1) or artifacts (class 0), continually adjusting weights to reduce the loss function and boost predictive accuracy. The model’s performance was assessed using various metrics:

Precision: The ratio of correctly identified *E. vermicularis* eggs (class 1) to all class 1 predictions.

Recall: The fraction of correctly identified *E. vermicularis* eggs (class 1) out of the total actual *E. vermicularis* eggs.

Accuracy: The overall rate of correct predictions made by the model.

F1-score: The harmonic mean of precision and recall.

Additionally, we plotted an ROC curve and calculated the ROC-AUC. The ROC-AUC provides a single metric summarizing the model’s overall performance, where 1.00 is ideal and 0.5 indicates performance equivalent to random guessing.

### 2.5. Object Detection and Bounding Box

The field of object detection has significantly advanced due to breakthroughs in ML, particularly in enhancing the precision and efficiency of bounding box predictions for identifying objects in images. To address the common issue of inaccurate bounding boxes, which can significantly impair object detector performance, the Object-Aware Multiple-Instance Learning (OA-MIL) method has been introduced. This approach enhances localization by leveraging classification accuracy, demonstrating effectiveness on both synthetic and real noisy datasets [14,20]. This method ensures that object detection remains accurate and robust despite data imperfections, improving reliability in real-world applications.

Finally, the Advanced IoU loss function addresses the shortcomings of existing loss functions in bounding box regression. By focusing on overlap area, distances, and side lengths, this function has shown improved accuracy, ensuring that object detection models are more precise and effective [21].

In our research, we have developed a comprehensive approach to enhance the accuracy and efficiency of object detection systems, specifically targeting the detection of small-scale objects such as *E. vermicularis* eggs. This methodology employs a sequence of image preprocessing and analysis steps facilitated by the OpenCV library and a trained CNN model. Here is a detailed breakdown of the methodology we employ (Figure 1A):

#### 2.5.1. Image Reading and Preprocessing

− Loading images: Each image is imported into our system using OpenCV’s cv2.imread() function. This step is essential to ensure the images are accurately loaded into the pipeline as arrays, preserving their integrity and original format.
− Image conversion: To simplify the processing, images are transformed to grayscale using cv2.cvtColor (image, cv2.COLOR_BGR2GRAY). This transformation reduces the data’s dimensionality, emphasizing textural and shape-based features, which are more significant for our task than color.
− Resizing images: Uniformity among input data is critical for the CNN’s performance. Images are resized to a standard size (e.g., 256 × 256 pixels) using cv2.resize(), meeting the model’s input specifications and ensuring consistency across all data.

#### 2.5.2. Object Detection and Analysis

− Initialization: Key parameters, such as bounding box sizes and step sizes, are set up. These parameters are essential for defining the sliding window mechanism utilized in our object detection approach.
− Patching and searching: This method involves extracting patches from the original image, each resized to 370 × 370 pixels with a step size of 50 pixels. These patches are then processed by the pre-trained CNN to predict the presence of *E. vermicularis* eggs.
− Heatmap generation: A heatmap is generated based on the model’s predictions, highlighting areas with scores above a set threshold (e.g., 0.8). This indicates potential *E. vermicularis* egg locations within the image.
− Object annotation: Areas identified with high confidence are marked with bounding boxes and automatically annotated by the model, indicating the detected regions of interest within the image.
− Output analysis: The results are compiled into two separate outputs: a heatmap showing the predicted object locations and the original image annotated with bounding boxes, class, and confidence level around the detected objects.

#### 2.5.3. Evaluation

To evaluate the effectiveness of our object detection system, we employ the IoU metric. This metric measures the overlap between the bounding boxes predicted by our model and those outlined by medical experts, providing a reliable indication of the model’s accuracy in identifying *E. vermicularis* eggs. By setting an IoU threshold, typically between 0.5 and 0.9, we classify detections as true positives or false positives. This systematic approach not only enhances the precision of detecting *E. vermicularis* eggs but also holds promise for broader applications in medical diagnostics and environmental monitoring, where precise object detection is critical.

## 3. Results

### 3.1. Outcomes of Image Recognition

**Figure 4:**
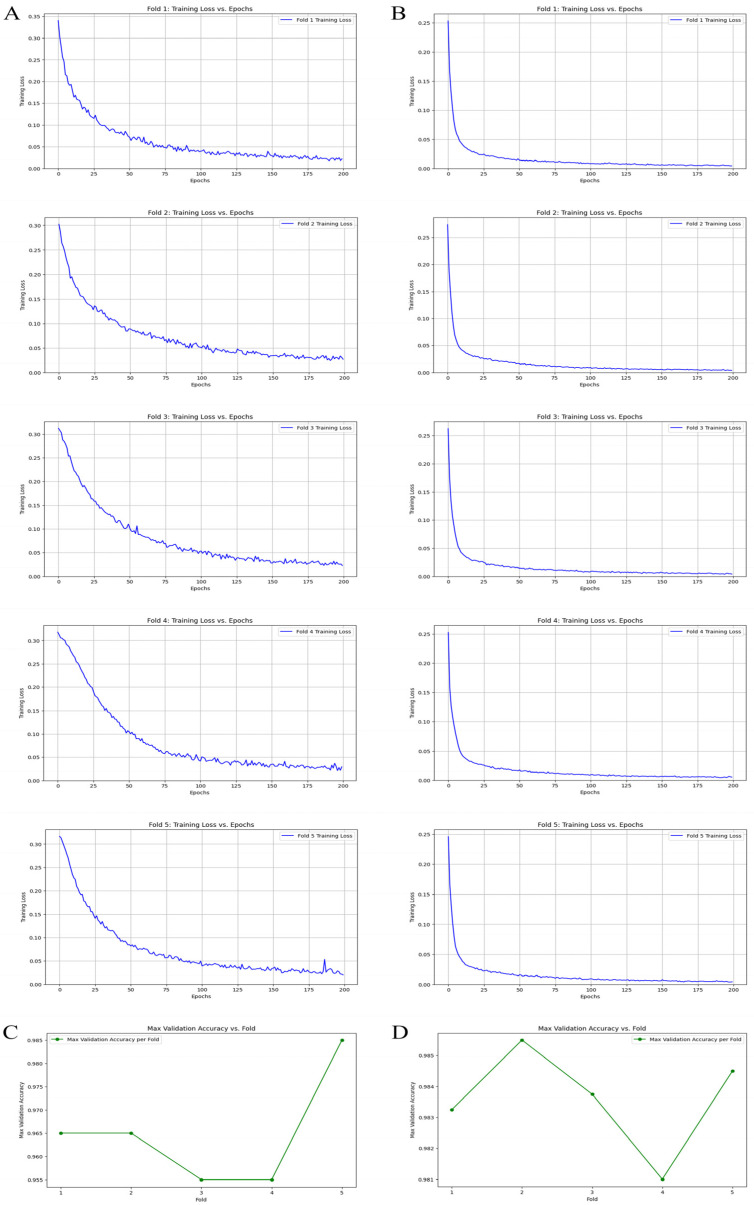
Outcomes of training and validating machine learning model. Results of five-fold cross-validation training loss. (**A**) Non-augmented image dataset. (**B**) Augmented image dataset. Relationship between prediction accuracy and number of folds used in cross-validation for image datasets. (**C**) Non-augmented image dataset. (**D**) Augmented image dataset.

During the model training and validation process of an ML model, a detailed examination of the results from a five-fold cross-validation reveals intriguing insights. As the model undergoes training, we observe the training loss for both non-augmented and augmented image datasets. Figure 4A,B clearly illustrate that the non-augmented image dataset experiences a significantly higher training loss. This heightened loss indicates that the model encounters greater difficulty in learning from the non-augmented data. The lack of diversity and variation in the non-augmented images likely contributes to this challenge, as the model struggles to generalize from such a homogeneous dataset. Conversely, the augmented image dataset, enriched with various transformations such as rotations, flips, and color adjustments, exhibits a lower training loss. This suggests that the model benefits from the increased variability, allowing it to learn more robust features and patterns.

Furthermore, when we delve into the relationship between prediction accuracy and the number of folds, a stark contrast emerges. Figure 4C,D highlight that the non-augmented image dataset shows significant instability in its prediction accuracy across different folds. The accuracy rates fluctuate considerably, reflecting the model’s inconsistent performance and vulnerability to the peculiarities of each fold.

In contrast, the augmented image dataset demonstrates remarkable stability in its prediction accuracy. The accuracy rates remain consistently high across all folds, underscoring the model’s improved generalization capabilities. This stability is a direct result of the augmented dataset’s ability to provide a more comprehensive representation of the data distribution, thereby enhancing the model’s robustness.

In summary, the five-fold cross-validation results underscore the critical importance of data augmentation in the training process of ML models. By incorporating augmented data, we not only reduce training loss but also achieve more stable and reliable prediction accuracy, ultimately leading to better-performing models.

**Figure 5:**
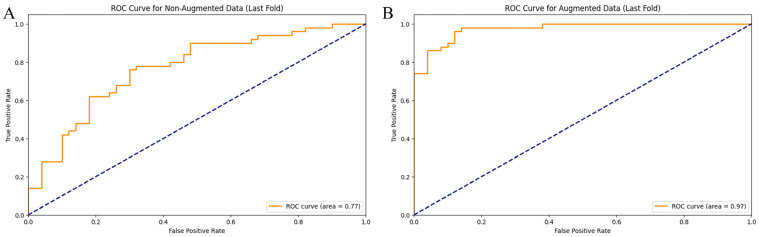
Receiver operating characteristic (ROC) curves for binary classification model trained on two different datasets. (**A**) Non-augmented image dataset. (**B**) Augmented image dataset. The orange line indicates the correct positives and misclassified negatives, highlighting the model’s class distinction ability. The blue dashed line indicates an AUC value of 0.5, suggesting no discriminative power.

The analysis of our CNN model for detecting *E. vermicularis* eggs revealed significant insights into the critical impact of data augmentation on model performance. Initially trained on unaltered data, the model achieved an accuracy, precision, recall, and F1-score of 71% (Table 1). These metrics provided a baseline to assess the effect of data augmentation. When we applied techniques such as rotations, noise addition, filtering, and sharpening, the model’s performance saw a remarkable improvement, with accuracy, precision, recall, and F1-score reaching 90%. This considerable enhancement underscores the importance of a diverse training dataset for developing reliable diagnostic tools, especially in medical image analysis, where variability in sample appearance can greatly affect diagnostic accuracy. Further validation using a rigorous five-fold cross-validation process demonstrated increased model stability and generalization. Figure 5 shows the ROC-AUC score, which measures the model’s ability to distinguish between the presence and absence of eggs, improved from 0.77 without augmentation to 0.97 with augmented data. This substantial improvement highlights the model’s enhanced discriminative ability, vital for accurate medical diagnostics.

### 3.2. Results of Image Identification

**Figure 6:**
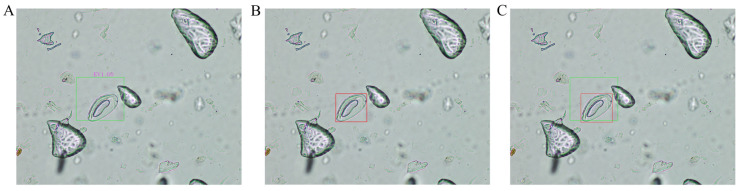
A detailed comparison between the object detection results of a highly trained machine learning model, Xception, and the annotations made by expert medical staff on the microscopic images. (**A**) The objects detected by the Xception model, highlighted by green bounding boxes. (**B**) The annotations made by a parasitology expert, indicated by red bounding boxes. (**C**) A combined view, displaying both the expert annotations (red bounding boxes) and the model’s predictions (green bounding boxes).

In our comparative evaluations, the Xception model (Table 1), trained on the augmented dataset, outperformed others with a 99% success rate across all metrics, including accuracy, precision, recall, and F1-score, and an average IoU of 0.6136 (Table 2 and Figure 6), indicating high precision in localizing and identifying *E. vermicularis* eggs. High IoU scores are crucial for reliable object detection in diagnostics. Additionally, comparisons included advanced architectures like MobileNet, EfficientNetB1, DenseNet121, InceptionV3, and ResNet50, all of which showed high performance with the augmented data, despite variations in complexity and computational demands. These findings validate our CNN model’s effectiveness and place it within the broader context of current DL solutions, emphasizing its efficiency with a smaller size and lower computational cost. The results of our study illustrate the profound impact of data augmentation in enhancing the performance and reliability of CNN models for microscopic detection of parasitic eggs. By expanding the dataset with augmented images, the model was exposed to a wider range of scenarios, significantly improving its ability to generalize from training data to real-world diagnostic challenges. This is especially crucial in medical imaging, where sample appearance variability can significantly affect diagnosis accuracy. These findings support the ongoing development and application of CNNs in parasitology, highlighting the potential for these technologies to transform diagnostic practices by increasing accuracy, reducing time, and potentially lowering the costs associated with traditional microscopic examinations.

## 4. Discussion

*Enterobius vermicularis*, commonly referred to as pinworm, is a parasitic nematode that is notorious for its high prevalence worldwide, particularly among children. This prevalence is notably higher in settings where close contact is common, such as schools, daycare centers, and households with young children. The ease of transmission through direct contact or contaminated surfaces makes children the most commonly affected group, often leading to widespread outbreaks in these communal environments. Traditionally, the detection of *E. vermicularis* has relied on methods such as the scotch tape test or direct fecal smear examinations. While these techniques are simple and widely used, they often suffer from low sensitivity and can be inconvenient for patients and healthcare providers alike. These traditional diagnostic methods frequently result in false negatives, leading to underdiagnosis and inadequate treatment, thereby perpetuating the cycle of infection and transmission. This limitation in detection poses significant challenges in controlling the spread of the parasite, particularly in endemic areas. In contrast, molecular techniques, especially polymerase chain reaction (PCR) assays, have emerged as powerful tools for the detection of *E. vermicularis*. PCR-based methods, including nested PCR, have demonstrated superior sensitivity and specificity in comparison to traditional diagnostic techniques. For example, a nested PCR assay targeting *E. vermicularis* has shown an impressive sensitivity of 88.9% and a specificity of 100%, significantly enhancing the accuracy of diagnosis and enabling better management of the infection [22]. The ability of PCR to detect even small amounts of parasite DNA in clinical samples makes it a highly effective tool for identifying infections that might otherwise be missed. Overall, while molecular methods like PCR represent a significant advancement in the detection of *E. vermicularis*, the challenges of cost and technical complexity underscore the need for further innovation.

Our research leverages the gold standard method for *E. vermicularis* detection, specifically the collection of *E. vermicularis* eggs using the scotch tape technique. This method provides a reliable basis for accurate detection, ensuring the quality of our dataset. To further enhance the robustness of our model, we employed four distinct filters, each designed to simulate different real-life imaging conditions. These filters not only represent various environmental factors but also serve to augment our image dataset, thereby improving the training process and overall performance of our model. No previous studies have emphasized investigating the diagnostic effectiveness of the CNN model in human enterobiasis. This study successfully demonstrated the application of CNNs in automating the detection and classification of *E. vermicularis* eggs. Despite variations in color, light intensity, clarity, egg morphology, and artifacts in the dataset, we demonstrated the effectiveness of using a CNN to detect *E. vermicularis* eggs in scotch tape test samples. Utilizing DL for diagnosing *E. vermicularis* infections aligns with the latest advancements in these fields, aiming to enhance the diagnostic precision and understanding of its epidemiology and clinical effects [23]. This approach not only improves diagnostic accuracy but also reduces the need for extensive human resources and time, offering a cost-effective and scalable solution for clinical practice compared to traditional diagnostic methods and molecular techniques. This study illustrates the impact of data augmentation in enhancing the effectiveness of CNNs for accurately detecting *E. vermicularis* eggs in children’s scotch tape test samples. Through thorough methodological strategies, including extensive data augmentation and robust cross-validation techniques, our CNN model demonstrated notable improvements in precision, reliability, and versatility across various diagnostic environments. This research represents a significant advancement in parasitological diagnostics, combining sophisticated ML methods with traditional microscopic examination to deliver more dependable, efficient, and accessible diagnostic services [22,24]. When comparing our CNN model with established models like MobileNet, EfficientNetB1, and Xception, our approach demonstrated competitive performance. Despite its smaller size, our CNN model maintained high diagnostic accuracy, highlighting the potential for developing lightweight models that do not require extensive computational resources. The exceptional performance of the Xception model, with nearly perfect metrics, further demonstrates the capabilities of advanced DL architectures in medical image analysis. The implications of this study extend beyond detecting *E. vermicularis*. The methodologies and findings can be applied to detecting other parasitic infections where a quick and accurate diagnosis is vital. By utilizing the scalability and adaptability of CNNs, similar techniques could be used to combat a range of infectious diseases, especially in low- and middle-income countries with high disease prevalence and limited diagnostic capabilities [24]. This paves the way for more efficient and effective diagnostic procedures, which could ultimately lead to improved disease treatment and sustainable control programs. However, the practical application of such advanced diagnostic tools in clinical settings must be approached cautiously. This requires comprehensive validation studies and ongoing monitoring of performance metrics to meet clinical standards in future work. This step was critical to ensuring that the model not only functions well under controlled laboratory environments but also maintains its accuracy and reliability when deployed in actual clinical settings. By simulating real-world scenarios, we were able to identify potential challenges and refine our approach to better align with the complexities encountered in everyday diagnostic processes. Given the high stakes of accurate diagnosis, particularly in cases of enterobiasis, we strongly recommend that laboratory staff re-examine all cases initially reported as negative using traditional microscopic methods for final confirmation. This re-examination is crucial because it mitigates the risk of false negatives, ensuring that no infections are overlooked. The microscopic confirmation serves as a safeguard, enhancing overall diagnostic reliability and maintaining high standards of clinical accuracy.

In future attempts, we plan to apply advanced model learning techniques and data generation methods to improve performance continuously. These techniques include the use of synthetic data to augment the training set as well as iterative learning algorithms to refine the model’s predictive capabilities. By incorporating these cutting-edge approaches, we aim to ensure that our model remains robust, adaptable, and capable of delivering high accuracy in diverse clinical and field environments. Our research also holds significant potential for expanding into telehealth applications, which could dramatically improve access to healthcare services in rural and underserved areas. Integrating our model into telehealth platforms can facilitate remote diagnosis of enterobiasis, allowing patients in remote locations to receive timely and accurate assessments without the need for direct access to specialized laboratory facilities. This application not only improves accessibility but also contributes to the broader goal of reducing healthcare disparities.

## Figures and Tables

**Table 1:** A comparison of the results from the conventional and the proposed models, including attributes, data performance metrics, and file size.

Models	Augmented Data	Accuracy	Precision	Recall	F1-Score	ROC-AUC	File Size (KB)
Proposed CNN	No	0.71	0.71	0.71	0.70	0.77	2804
Proposed CNN	Yes	0.90	0.90	0.90	0.89	0.97	2804
MobileNet	Yes	0.96	0.96	0.96	0.95	1.00	50,439
EfficientNetB1	Yes	0.89	0.90	0.89	0.88	0.94	93,516
DenseNet121	Yes	0.97	0.97	0.97	0.96	1.00	96,155
Xception	Yes	0.99	0.99	0.99	0.99	1.00	269,365
InceptionV3	Yes	0.98	0.98	0.98	0.97	1.00	281,301
Resnet50	Yes	0.99	0.99	0.99	0.99	1.00	375,244

**Table 2:** The results include the intersection-over-union (IoU) range and the image count by Xception.

IoU	Image Count	Average IoU	%Average IoU
<0.5	3	0.6136	61.36
>0.5	95	0.3241	32.41

## Data Availability

Our publicly accessible dataset of *E. vermicularis* egg images is available at Figshare (https://doi.org/10.6084/m9.figshare.26266028.v2, accessed on 22 August 2024). The codes are available at the following GitHub repository: https://github.com/natthanaic/DevMLEV (accessed on 22 August 2024).

## References

[1] Lashaki E.K., Mizani A., Hosseini S.A., Habibi B., Taherkhani K., Javadi A., Taremiha A., Dodangeh S. (2023). Global prevalence of enterobiasis in young children over the past 20 years: A systematic review and meta-analysis. Osong Public Health Res. Perspect..

[2] Wongsaroj T., Nithikathkul C., Reungsang P., Royal L., Nakai W., Krailas D., Ramasoota P. (2012). Geographic information of helminthiasis in Thailand. Int. J. Geoinform..

[3] Sung J.F., Lin R.S., Huang K.C., Wang S.Y., Lu Y.J. (2001). Pinworm control and risk factors of pinworm infection among primary-school children in Taiwan. Am. J. Trop. Med. Hyg..

[4] Wendt S., Trawinski H., Schubert S., Rodloff A.C., Mössner J., Lübbert C. (2019). The diagnosis and treatment of pinworm infection. Dtsch. Arztebl. Int..

[5] Vaisman A., Linder N., Lundin J., Orchanian-Cheff A., Coulibaly J.T., Ephraim R.K., Bogoch I.I. (2020). Artificial intelligence, diagnostic imaging and neglected tropical diseases: Ethical implications. Bull. World Health Organ..

[6] Kumar S., Arif T., Alotaibi A.S., Malik M.B., Manhas J. (2023). Advances towards automatic detection and classification of parasites microscopic images using deep convolutional neural network: Methods, models and research directions. Arch. Comput. Methods Eng..

[7] Naing K.M., Boonsang S., Chuwongin S., Kittichai V., Tongloy T., Prommongkol S., Dekumyoy P., Watthanakulpanich D. (2022). Automatic recognition of parasitic products in stool examination using object detection approach. PeerJ Comput. Sci..

[8] Pedraza A., Ruiz-Santaquiteria J., Deniz O., Bueno G. Parasitic egg detection and classification with transformer-based architectures. Proceedings of the 2022 IEEE International Conference on Image Processing (ICIP).

[9] Delas Peñas K.E., Villacorte E.A., Rivera P.T., Naval P.C. Automated detection of helminth eggs in stool samples using convolutional neural networks. Proceedings of the 2020 IEEE Region 10 Conference (TENCON).

[10] Ruiz-Santaquiteria J., Pedraza A., Vallez N., Velasco A. Parasitic egg detection with a deep learning ensemble. Proceedings of the 2022 IEEE International Conference on Image Processing (ICIP).

[11] Rocha M., Claro M., Neto L., Aires K., Machado V., Veras R. (2023). Malaria parasites detection and identification using object detectors based on deep neural networks: A wide comparative analysis. Comput. Methods Biomech. Biomed. Eng. Imaging Vis..

[12] Goceri E. (2023). Medical image data augmentation: Techniques, comparisons and interpretations. Artif. Intell. Rev..

[13] Higuchi K., Mizuhashi T., Matulic F., Igarashi T. Interactive generation of image variations for copy-paste data augmentation. Proceedings of the CHI′23: CHI Conference on Human Factors in Computing Systems.

[14] Liu X., Ono K., Bise R. Mixing data augmentation with preserving foreground regions in medical image segmentation. Proceedings of the 2023 IEEE 20th International Symposium on Biomedical Imaging (ISBI).

[15] Thanchomnang T., Chaibutr N., Maleewong W., Janwan P. (2024). Automatic detection of *Opisthorchis viverrini* egg in stool examination using convolutional-based neural networks. PeerJ.

[16] Peng Y., Meng Z.Q., Yang L.A. (2023). Image-to-image translation for data augmentation on multimodal medical images. IEICE Trans. Inf. Syst..

[17] Ialithabhavani B., Krishnaveni G., Malathi J. A comparative performance analysis of different machine learning techniques. Proceedings of the International Conference on Computer Vision and Machine Learning.

[18] Cowley H.P., Natter M., Gray-Roncal K., Rhodes R.E., Johnson E.C., Drenkow N., Shead T.M., Chance F.S., Wester B., Gray-Roncal W. (2022). A framework for rigorous evaluation of human performance in human and machine learning comparison studies. Sci. Rep..

[19] De Ranasinghe I.M.M.P., Munasinghe L. (2023). Comparison of performances of ML-Algorithms in the estimation of the execution time of non-parallel Java programs. J. Sci. Univ. Kelaniya.

[20] Yao Y., Cheng G., Wang G., Li S., Zhou P., Xie X., Han J. (2022). On improving bounding box representations for oriented object detection. IEEE Trans. Geosci. Remote Sens..

[21] Nguyen H.S.H., Tran T.H.G., Tran D.K., Pham D.D., Anh N.L., Koh S.J., Nguyen T.D.L., Lloret J., Nguyen T.T. (2022). An advanced IoU loss function for accurate bounding box regression. Intelligent Systems and Networks.

[22] Ummarino A., Caputo M., Tucci F.A., Pezzicoli G., Piepoli A., Gentile A., Latiano T., Panza A., Cala N., Ceglia A.P. (2022). A PCR-based method for the diagnosis of *Enterobius vermicularis* in stool samples, specifically designed for clinical application. Front. Microbiol..

[23] Lee Y.W., Choi J.W., Shin E.H. (2021). Machine learning model for diagnostic method prediction in parasitic disease using clinical information. Expert Syst. Appl..

[24] Zafar A., Attia Z., Tesfaye M., Walelign S., Wordofa M., Abera D., Desta K., Tsegaye A., Ay A., Taye B. (2022). Machine learning-based risk factor analysis and prevalence prediction of intestinal parasitic infections using epidemiological survey data. PLoS. Negl. Trop. Dis..

